# Variable Innervation of the Triceps Brachii: Anatomical and Clinical Perspectives in a Narrative Review

**DOI:** 10.7759/cureus.100537

**Published:** 2025-12-31

**Authors:** Harpreet Kaur Mohel, Gitanjali Khorwal, Rahul Sharma, Bhamini Sharma

**Affiliations:** 1 Anatomy, All India Institute of Medical Sciences, Rishikesh, Rishikesh, IND

**Keywords:** axillary nerve, distal triceps repair, radial nerve branch, the cadaveric study, ulnar nerve (un)

## Abstract

Traditional anatomical texts describe the triceps brachii as receiving motor innervation exclusively from branches of the radial nerve. However, growing morphological, electrophysiological, and clinical evidence demonstrates notable variability in its neural supply, particularly to the long and medial heads of the muscle. To clarify these inconsistencies, a narrative review was conducted using PubMed, Scopus, and Google Scholar with search terms related to triceps innervation and nerve variations. After screening 930 records, 27 studies met the inclusion criteria. The articles were reviewed and presented in a table format, comprising original human cadaveric and clinical data. Relevant information on study design, sample size, and specific innervation patterns was extracted and organised, with selection illustrated using a Preferred Reporting Items for Systematic Reviews and Meta-Analyses (PRISMA) flowchart. The compiled evidence from cadaveric dissections and clinical case reports reveals frequent deviations from the classic pattern. Numerous studies report that the axillary nerve contributes to the long head, with prevalence ranging from isolated findings to over 35% in certain populations. The medial head has been shown in several investigations to receive branches from the ulnar nerve, and rare cases describe musculocutaneous nerve fibres replacing distal radial innervation. These observations underscore a more complex neural architecture than typically depicted. Recognising such variations is crucial for academic accuracy and clinical practice. It facilitates improved interpretation of nerve injuries, informs surgical and nerve transfer procedures, and supports more precise electrophysiological assessment. Collectively, the evidence highlights the need to update standard anatomical descriptions and encourages continued research into the functional implications of these innervation patterns.

## Introduction and background

The triceps brachii has three heads of origin and is a muscle situated in the posterior compartment of the arm. All three heads converge into a common tendon, which is inserted into the olecranon process of the ulna [1]. Triceps muscles are primarily innervated by the radial nerve [1,2], coming from the posterior cord of the brachial plexus, and their function is chiefly to extend the forearm at the elbow joint, also acting as a synergist in upper limb movements, particularly during shoulder abduction and stabilisation. During development, around day 16, the intraembryonic mesoderm differentiates into paraxial mesoderm, which segments into somites. During embryonic development, the musculature of the upper limb originates from hypaxial cells of the dermomyotome derived from cervical and upper thoracic somites. These myogenic precursor cells migrate into the limb bud during the fifth week of development and segregate into dorsal (extensor) and ventral (flexor) muscle masses. The triceps brachii develops from the dorsal muscle mass of the upper limb, which is innervated by the posterior divisions of the ventral rami. This developmental organisation explains the classical radial nerve innervation of the triceps brachii and provides an embryological basis for observed variations in its nerve supply when there are alterations in muscle differentiation or nerve branching patterns [2].

Segmental innervation of the triceps muscles is mainly derived from C6, C7, and C8 fibres for the long head, lateral head, and medial head, respectively [1]. At the same time, various studies have reported variations in this classical innervation pattern. Long head of triceps brachii (LHT) is seen affected in axillary nerve injuries [3], while surgical observations have identified branches from the ulnar nerve supplying portions of muscle [4]. Cadaveric investigations have further demonstrated that the long and medial heads may receive additional innervation from axillary and ulnar nerves, respectively [5-7].

Axillary nerve injuries may result from trauma, iatrogenic causes, brachial plexus lesions, or quadrilateral space syndrome [6,8]. Isolated axillary nerve injury, often following shoulder dislocation or plexus trauma, compromises the deltoid and teres minor (TM), leading to weakness in shoulder abduction and external rotation [9-11]. Indeed, up to 95% of brachial plexus injuries involve restricted shoulder mobility [12].

From a reconstructive perspective, the long head of the triceps brachii is frequently utilised as a free functional muscle graft or as a donor of motor branches in nerve transfer procedures [13]. Its effectiveness in restoring shoulder abduction and external rotation relies on its motor innervation, which may vary from the classical radial nerve supply to include contributions from the axillary nerve [14]. Similarly, the medial head may receive motor branches from the ulnar or ulnar collateral nerve, allowing its selective use in reinnervation strategies. Awareness of these innervation variations is essential for selecting appropriate donor nerves, preventing iatrogenic injury, and optimising postoperative motor re-education and functional recovery following axillary or posterior cord injuries [4].

This review aims to present the innervation of triceps brachii beyond its traditional radial nerve supply. Recognising such variations holds substantial clinical relevance, aiding in the prevention of iatrogenic nerve injuries, improving diagnostic precision, and guiding nerve reconstruction, electrophysiological assessment, and rehabilitation. Furthermore, this knowledge enhances anatomical and surgical education concerning upper limb neuroanatomy.

## Review

Material & methods

**Figure 1:**
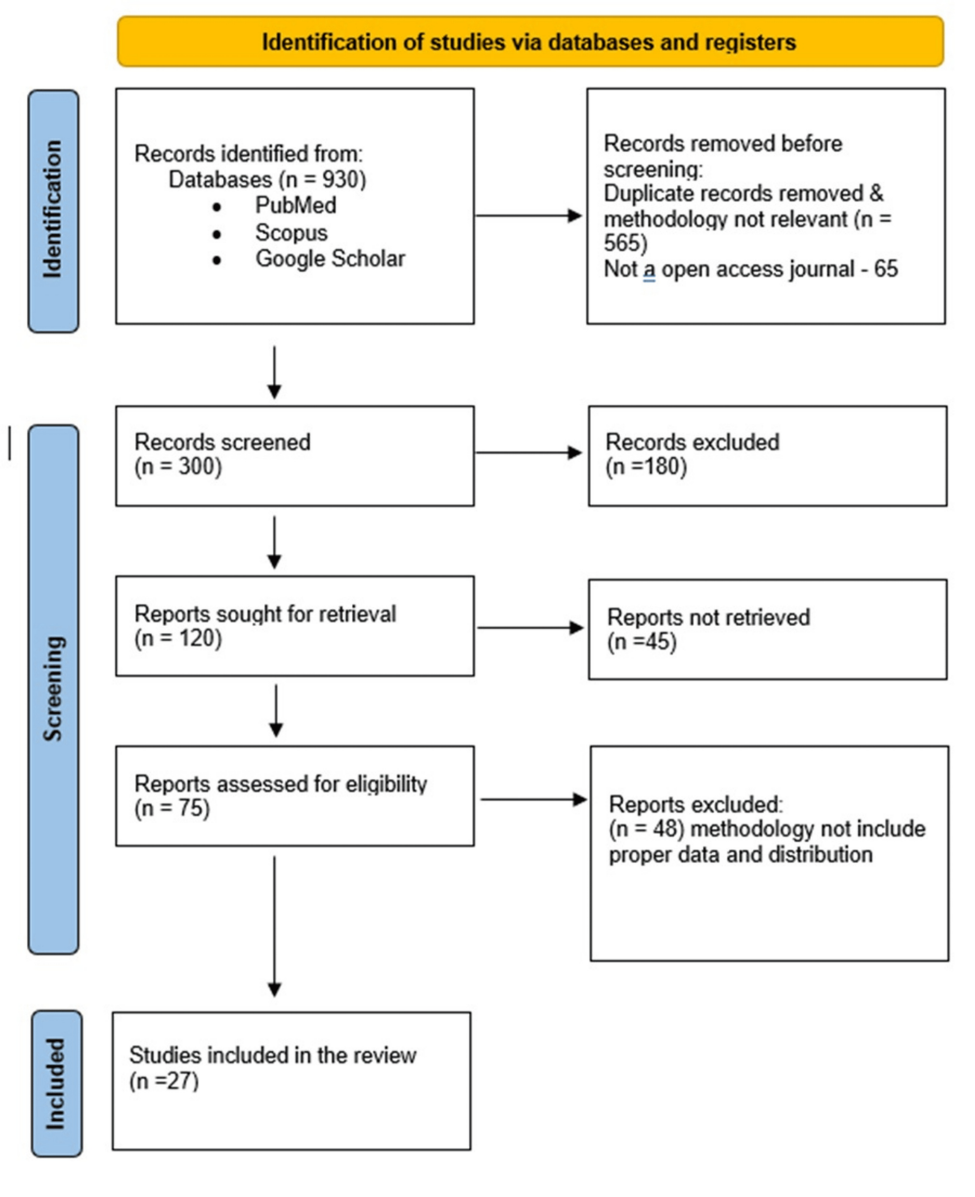
Search and selection of studies for the narrative review shown in the given chart.

A narrative review was conducted to examine the innervation patterns of the triceps brachii muscle, including both cadaveric dissection studies and clinical observational reports. Through thorough searches of electronic databases, such as PubMed, Scopus, and Google Scholar, pertinent studies were identified. Combinations of the keywords used were triceps brachii, innervation, radial, axillary, ulnar nerve, and nerve variation. Reference lists of retrieved publications were also screened to identify additional relevant studies. Search and selection of studies for the narrative review are presented in a chart for better schematic illustration, as shown in Figure 1.

Inclusion and exclusion criteria

Studies were included if they (1) provided original data on the nerve supply of the triceps brachii, (2) involved human cadaveric or clinical specimens, and (3) clearly described the specific innervation of the long, lateral, or medial heads. Reviews without primary data, animal studies, and case reports with insufficient anatomical detail were excluded.

Data extraction and analysis

Twenty-seven studies out of 930 met the inclusion criteria. Data were extracted on sample size, study type, and observed innervation patterns for each head of the triceps. The findings were tabulated and grouped by muscle head to facilitate comparison and synthesis (Figure 1).

Observation and review

The narrative synthesis of the reviewed literature revealed distinct and variable patterns of innervation among the different heads of triceps brachii.

For LHT, 15 studies, as shown in Table 1, comprising a total of 336 specimens, were analysed. Among these, 55 specimens demonstrated innervation by the axillary nerve, suggesting that while the radial nerve remains the primary source of innervation, an accessory or variant axillary nerve contribution may occur in a noteworthy proportion of cases.

**Table 1:** Shows cadaveric and clinical findings of the long head of triceps brachii (LHT) muscle's innervation. UE: upper extremity; UL: upper limb.

Author/year	Study type	Research approach	Finding
Aszmann et al. 1996 [6]	25 Cadaveric study	Cadaveric investigation of the shoulder joint innervation	In 7 (28%) specimens, the tendinous insertion of LHT is supplied by a small branch from the axillary nerve
De Seze et al. 2004 [7]	29 Cadaveric study	Cadaveric and surgical investigation of the triceps brachii's long head nerve innervation	In 13 cases, the motor branch of LHT is from the axillary nerve, in five cases, the motor branch is from the terminal division of the posterior cord, in two cases, the branches are from the posterior cord before its division to LHT
De Seze et al. 2004 [7]	15 Surgical dissections		Eleven cases branch from the axillary nerve to LHT, four cases branch from the posterior cord to LHT
Bertelli et al. 2007 [9]	10 Cadaveric study	Investigating the axillary nerve for the surgical repair	One specimen, posterior branch of the axillary nerve perforates the LHT muscle
Al-Meshal et al. 2013 [15]	25 Cadaveric study	Cadaveric investigation of the triceps brachii muscle's nerve and its utilization in nerve transfer	In all the cases, the LHT brachii muscle is supplied only by the radial nerve
Erhardt et al. 2017 [16]	22 Cadaveric study	Cadaveric investigation to explore the axillary nerve supply to LHT	Eight specimens were innervated by the radial nerve, and out of 14 specimens, 11 have dual innervation from the axillary and radial nerve, and three specimens are innervated exclusively by the axillary nerve
Chaware et al. 2018 [17]	36 Cadaveric study	Cadaveric investigation to identify the triceps brachii muscle innervation	Exclusive axillary nerve supply to LHT was observed in 1 (3%) specimen. One specimen has dual nerve supply from the radial and axillary nerves, and in the rest of the specimens, nerve supply to LHT is by the radial nerve
Wade et al. 2018 [18]	27 Cadaveric study review of literature	Cadaveric investigation of the posterior cord and the triceps brachii muscle	In all specimens, nerve supply to LHT muscle is exclusively by the radial nerve
Anitha et al. 2019 [19]	60 Cadaveric study	Cross-sectional study to investigate the branching pattern of the axillary nerve and its variation	In right side of one cadaver, muscular branch of posterior division of axillary nerve supplies LHT muscle
Mehta et al. 2020 [20]	30 Cadaveric study	To assess the prevalence of the axillary nerve contribution to the LHT	In 8 (26.6%) dissected UE axillary nerve innervate the LHT muscle and according to branching pattern was classified into three types
Peer et al. 2024 [21].	52 Cadaveric study	Study the occurrence of variant branches to the triceps brachii	Six specimens LHT are innervated by the axillary nerve, and the rest of the 46 specimens are innervated by the radial nerve
McClelland et al. 2008 [22]	Single clinical case report	Decompressing the axillary nerve	In nerve conduction study, denervation changes in (L) deltoid, fasciculation affecting triceps brachii and deltoid muscle with impingement of the axillary nerve
Sawant 2015 [23]	Single cadaveric case report study	During routine dissection for the first year axillary nerve branch to LHT was observed	In a 70-year-old male cadaver, the nerve supply to the LHT muscle was observed from axillary nerve
Singh et al. 2020 [24]	Single clinical case report (neurosurgery)	Intraoperative aberrant nerve observed	During the surgery of 21-year-old boy, it was identified that the LHT muscle was supplied by a branch from the axillary nerve
Dasegowda et al. 2021 [25]	Single cadaveric case report (review of literature)	During dissection for the first year of MBBS, the axillary nerve to the LHT was observed	In a 60-year-old male cadaver, the right limb showed the innervation to the LHT brachii muscle is from anterior branch of axillary nerve
Joy et al. 2020 [26]	Single cadaveric case report	During routine posterior arm dissection, a variant nerve supply to LHT was observed	Dissecting the quadrilateral area of the arm on the right side, it was found that the axillary nerve pierced the LHT, and LHT was not supplied by any branch from the radial nerve. Same was observed on the left side

For the medial head of the triceps (MHT), 11 studies represented in Table 2, including 318 specimens, were reviewed. Ulnar nerve innervation of MHT was identified in 64 specimens, while a collateral branch of the radial nerve supplying this head was reported in 78 cases. In addition, a communicating branch between the ulnar and radial nerves was documented in 11 specimens, reflecting the presence of complex neural interconnections in the posterior arm compartment.

**Table 2:** Shows cadaveric and clinical findings of the medial head of triceps brachii muscle's (MHT) innervation. UCN: ulnar collateral nerve.

Author/year	Study type	Research approach	Finding
Bekler et al. 2009 [4]	18 Cadaveric study and histologic staining	Cadaveric investigation to determine the ulnar nerve branch to the triceps brachii's medial head	In 17 specimens, there is branch from the ulnar nerve to the MHT muscle and out of 17 in six cases accessory ulnar collateral branch was observed and above observation was confirmed histologically
Scogin et al. 2023 [27]	15 Cadaveric (foetal and six adult arms) study and IHC	Cadaveric investigation of triceps brachii muscle innervation	In 15 foetal upper limbs, MHT supplied by UCN
Pascual-Font et al. 2013 [28]	50 Cadaveric study	Investigating the ulnar nerve branch to the MHT	In 14 (28%), the ulnar nerve branch to MHT
Loukas et al. 2013 [29]	60 Cadaveric study	Investigating the ulnar nerve branch to the triceps brachii	The branch from ulnar nerve to heads of triceps muscle was observed in nine cases; predominant branch is to MHT; communication between ulnar nerve and radial nerve in 4 UL was observed; and in one of the limb communications between ulnar nerve branch and musculocutaneous nerve
Silva et al. 2017 [30]	35 Cadaver study	Cadaveric investigation of the triceps brachii muscle nerve supply	In five specimens, the branch from ulnar nerve to MHT and communicating branch were also found between the ulnar nerve and the radial nerve in one limb
Chaware et al. 2018 [17]	36 Cadaveric study	Cadaveric investigation to identify the triceps brachii muscle innervation	One (3%) had dual supply from ulnar nerve and radial nerve
Develi 2018 [31]	32 Cadaveric study	To investigate prevalence of ulnar nerve supply to triceps	In 43.8% MHT was innervated by ulnar nerve
Jain et al. 2019 [32]	68 Cadaveric study	To analyse the ulnar collateral nerve prevalence	In 57 specimens, UCN was observed to the MHT muscle
Miguel-Pérez et al. 2010 [33]	Single cadaveric case report	During routine dissection aberrant innervation to the triceps brachii was found	In a 79-year-old cadaver, motor branch to MHT from ulnar nerve was noted
Ajayi et al. 2012 [34]	Single cadaveric case report	During routine dissection incidentally connection between the nerve was found	Communicating branch between ulnar nerve and radial nerve was noted on both sides
Swamy et al. 2015 [35]	Single cadaveric report	During routine dissection of the medical students communication between nerves was found	Communicating branch was found between in ulnar and radial nerve and twig from these supplies to MHT

Comparison across studies indicates that variations in triceps innervation are most frequently associated with the medial head, whereas long and lateral heads display more consistent radial nerve supply. The increasing number of reports identifying ulnar nerve contributions to the medial head underscores a potential functional overlap and anatomical variability not always represented in standard anatomical descriptions. Such findings bear clinical significance, particularly in surgical procedures involving the posterior arm, nerve transfers, and electromyographic assessments, where awareness of these variants is essential to prevent iatrogenic injury and to optimise reconstructive outcomes.

Main outcomes

Routine dissection studies have reported that the LHT may receive a branch from the axillary nerve rather than the typical radial nerve supply [23,25]. Sawant observed in a 70-year-old male cadaver that the LHT of the right upper limb was innervated by a muscular branch of the posterior division of the axillary nerve, which also supplied the teres minor and deltoid muscles [23]. Similarly, Dasegowda et al. reported in a study on a 60-year-old male cadaver that the right LHT was supplied by the anterior division of the axillary nerve rather than the radial nerve [25]. In both studies, the left LHT retained the conventional radial nerve innervation. Additionally, a case report done by Scogin et al. described an 89-year-old male cadaver in which LHT was innervated by the axillary nerve on both sides of the upper limb [27].

Clinical observation also supports this variation, patients with loss of shoulder abduction movement in traumatic brachial plexus injury involving the C5-C6 root, quadrilateral space syndrome [7,22,24]. They found the association of weakness of the triceps brachii with it. In a case report conducted by McClelland et al., a study was done on a 26-year-old swimmer who presented with visible muscular twitching in his LHT of his left shoulder, neurophysiological test reveals denervation changes in the left deltoid with fasciculation affecting the deltoid and LHT, and after surgery, it was seen that fasciculations in triceps brachia’s long head has ceased [22]. Surgical and cadaveric studies by Singh et al. and De-Seze have confirmed these variations. Singh et al., during surgery, found abnormal innervation of the LHT muscle from the axillary nerve [24]. De-Seze et al. performed study into two groups a surgical dissection and cadaveric, during surgical procedure there is contraction of the LHT through the axillary nerve stimulation with a nerve stimulator and then found that in 11 cases motor branch arose from axillary nerve and in four cases from terminal division of posterior cord and in cadaveric dissection they state that motor branch of LHT arose from axillary nerve in 13 cases, from terminal division of posterior cord in five case and from posterior cord in two cases, in both groups motor branch to LHT did not arises from radial nerve [7].

Various studies have been conducted to determine the prevalence of the axillary nerve innervating the LHT [16,20,21]. Mehta et al. studied 30 upper extremities and found that the axillary nerve supplied LHT in eight upper limbs and classified the branching patterns of the axillary nerve into three different types. In type 1, the posterior branch of axillary nerve supplies LHT in four limbs (50%), in type 2, the branch to TM supplies LHT in three limbs (37.7%), and in type 3, a branch from the bifurcation of the anterior and posterior branches of axillary nerve supplies LHT in one limb (12.5%) [20]. Erhardt et al. studied 22 brachial plexuses. They observed a classic pattern that the LHT is supplied by the radial nerve in eight specimens, and a non-classic pattern that is innervation to LHT other than the radial nerve in 14 specimens, out of which 11 specimens have dual innervation that is from the axillary nerve and radial nerve, and three specimens were supplied by the axillary nerve only [16]. Peer et al. state that out of 52 cadaveric limbs, LHT is supplied by the radial nerve in 46 cadavers, and six are supplied by the axillary nerve [21].

On the contrary, studies all show innervation of the triceps brachii muscle only from the radial nerve [15,18]. Wade et al. performed cadaveric dissection on 27 upper limbs, and all 27 upper limbs are solely supplied by the radial nerve; no branch from the axillary nerve innervates LHT [18]. A study by Al-Meshal et al. noted that in all 22 cadavers, the radial nerve supplies three heads of the triceps brachii muscle [15].

However, Anitha et al. and Bertelli et al. studied the branching pattern of the axillary nerve. Bertelli et al. performed cadaveric dissection and surgical repair and found variation during dissection of the one cadaver, which shows that LHT was perforated by the posterior branch of the axillary nerve [9]. Anitha et al. found variation in one case of the right-sided female cadaver muscular branch of the posterior division of the axillary nerve, which gives a branch to LHT, which was also supplied by the radial nerve [19]. This study is similar to the survey done by Sawant [23]. Another study done by Aszmann et al. noted that a small branch from the muscular branch of the axillary nerve to the tendinous insertion of the triceps and to the adjacent capsular region in seven specimens [6].

Understanding these variations is essential during surgeries to prevent iatrogenic nerve injury. The LHT can be used as a free functional muscle graft, used as a donor muscle in restoring the function of the deltoid in upper brachial plexus injury.

Innervation of the Medial Head of the Triceps

Some studies show that MHT is supplied by the ulnar nerve and the ulnar collateral branch of the radial nerve [4,28,30,31]. Ulnar collateral branch of the radial nerve leaves the radial nerve at the level of the brachioaxillary angle and travels along the ulnar nerve [4].

Bekler et al. performed cadaveric dissection and histological staining on 18 upper limbs, and in 11 specimens, MHT is supplied by the main ulnar nerve, and in six specimens, MHT is supplied by the ulnar collateral branch, which is in approximation with the ulnar nerve, as confirmed histologically [4]. Jain et al. observed that the medial head of the triceps brachii muscle is supplied by the ulnar nerve branches in 14 cases; the left side presented 57.1% ulnar nerve innervation, while the right side revealed 42.9% [32]. According to a study by Loukas et al., the ulnar nerve branch to MHT in 14 of the 50 dissected arms was observed [29]. Another study by Develi revealed a branch from the ulnar nerve to the MHT in 5 (14.7%) of the dissected arms [31].

However, some studies observe that the triceps brachii was not supplied by the ulnar nerve [27,32]. Jain et al. observed that the ulnar collateral nerve in 57 (83.8%) cases innervates the MHT, and UCN can arise in the axilla, brachioaxillary angle, and in the arm; in no case ulnar nerve observed to triceps brachii muscle was observed [32]. A study by Pascual-Font et al. in 15 embryonic and foetal upper limbs, a branch from the radial nerve that is the ulnar collateral nerve, arises at the level of the axilla and runs towards the ulnar nerve [28]. These branches are closely related to the ulnar nerve sheaths in the upper part of the arms, but do not intersect with the nerve, and distally they enter the medial heads of the triceps brachii muscle, and no branch was given from the ulnar nerve to the medial head of triceps. The immunohistochemical analysis done on the adult sample revealed that the ulnar collateral nerve carries some motor and non-motor fibres. The study indicates that MHT is supplied entirely by the radial nerve and not by the ulnar nerve. Although certain nerve branches travel alongside the ulnar nerve, histological research reveals that they arise from the radial nerve.

Some of the studies revealed a dual pattern of innervation to the MHT. Miguel-Pérez et al. report that the motor nerve branch of the ulnar nerve to the MHT was seen in a 79-year-old female cadaver [33]. An anatomic communication found between the ulnar and radial nerves in a study done by Ajayi et al., on the right side, there was only one communicating branch, whereas the left side had many communicating twigs between the ulnar and radial nerves at the mid-humeral level [34]. However, innervation to the triceps from this was not reported. Similarly, Swamy et al. revealed a communicating branch between the radial and ulnar nerves, and a twig from this gives a branch to the MHT muscle [35].

Studies Done on All Three Heads of Triceps Brachii

Loukas et al. studied that three heads are supplied by the radial nerve, and the ulnar nerve also gives branches to all three heads. The lateral head is supplied by one branch, the long head is supplied by two branches, and the MHT receives eight branches [29]. Chaware et al. studied the upper limbs of 36 cadavers and found that MHT in one specimen was innervated by the radial nerve and ulnar nerve, and in four specimens, dual innervation from radial and axillary nerves to LHT was seen, and in one specimen, LHT was supplied by the axillary nerve branch [17].

A report by Yogesh et al. observed unilateral variation in which the musculocutaneous nerve replaced the distal part of the radial nerve [36]. Anatomic variation in the nerve supply to the triceps brachii muscle shows significant variability. Literature reveals that the axillary nerve also supplies the LHT; similarly, the ulnar nerve and the ulnar collateral branch innervate the MHT. Dual innervation can maintain muscle function and enhance the outcomes of nerve transfer and repair surgeries.

Discussion

Traditionally, dysfunction of the triceps brachii has been attributed primarily to radial nerve injury. However, cumulative evidence from cadaveric, clinical, and surgical studies demonstrates that the innervation of this muscle is not invariably radial. Variants involving contributions from the axillary nerve, ulnar nerve, or ulnar collateral nerve have been documented, thereby altering conventional interpretations of nerve injury patterns and associated muscle dysfunction.

Variant Innervation Patterns

Several cadaveric studies have specifically reported axillary nerve innervation of the long head of triceps brachii (LHT), suggesting this as the most frequently observed variation [16,20,21]. Less commonly, branches associated with the ulnar nerve have been described supplying the medial head of triceps brachii (MHT), as reported by Pascual-Font et al. and Silva et al. [28,30]. In such cases, dual innervation from the axillary and ulnar nerves may contribute to partial preservation of muscle function following isolated nerve injuries. These findings collectively support the presence of reproducible but infrequent anatomical variations that hold functional significance.

In contrast, several studies continue to demonstrate that the radial nerve supplies all heads of the triceps brachii [15,18]. Furthermore, some authors report that the ulnar collateral nerve does not provide independent branches to the muscle [27,32]. Pascual-Font et al., using foetal dissections and immunohistochemical techniques, confirmed that so-called ulnar collateral branches to the triceps originate from the radial nerve, despite their close anatomical relationship to the ulnar nerve [28]. These observations reinforce the radial nerve as the principal motor nerve of the triceps brachii, while acknowledging well-documented exceptions.

Clinical and Surgical Implications

Awareness of variant triceps innervation is clinically important for accurate diagnosis, electrophysiological interpretation, surgical planning, and rehabilitation in peripheral nerve injuries. The triceps brachii is widely used as a free functional muscle graft in upper limb reconstruction, rotator cuff injuries, chest wall defects, and contracture release following burns [13,23,37-39]. In nerve reconstruction, Bertelli et al. successfully utilised the long head branch of the axillary nerve for restoration of deltoid and teres minor function [9]. Additionally, the long and medial head branches of the triceps (modified Somsak technique) are employed in nerve transfer procedures to the anterior branch of the axillary nerve to restore shoulder abduction in upper brachial plexus and isolated nerve injuries [40].

The long head branch of the triceps is favoured due to its length, adequate motor fibre content, and minimal functional deficit following transfer, while acting synergistically in shoulder abduction and external rotation, facilitating postoperative motor re-education [9,24,41,42]. Variant innervation also has implications in sports medicine, where atypical weakness or unexpected recovery patterns may occur [43]. McClelland et al. reported triceps involvement in axillary nerve injury in a competitive swimmer, and Singh et al. documented aberrant nerve supply to the muscle [22,24]. Recognition of such variants may prevent misdiagnosis and inappropriate rehabilitation strategies in athletic populations.

Overall, these findings highlight that although the radial nerve supply remains the dominant pattern, awareness of anatomical variations in triceps brachii innervation is essential for accurate clinical assessment and optimisation of surgical and rehabilitative outcomes.

Limitations

(1) The study was based on cadaveric dissections, which allow precise visualisation of neural anatomy but cannot fully replicate in vivo conditions, as post-mortem tissue changes and the absence of nerve conduction or muscle activity may influence identification of fine branches and their functional relevance. (2) The single-centre design and limited sample size provide focused anatomical detail but may not capture the full extent of population-level variability in triceps brachii innervation, including potential ethnic differences. (3) The investigation relied on gross dissection, which is effective for mapping nerve courses but does not permit definitive differentiation between motor and sensory fibres; consequently, some branches--particularly those associated with the ulnar nerve--may represent collateral branches of the radial nerve rather than true ulnar motor innervation. (4) The absence of functional or clinical correlation allowed emphasis on anatomical patterns, but limits direct translation of these findings to clinical or functional outcomes. (5) Future studies employing multicentric samples and integrating anatomical, histological, and functional approaches are anticipated to clarify prevalence, confirm functional significance, and enhance the clinical applicability of these anatomical observations.

## Conclusions

The present review highlights the considerable variability in the innervation of the triceps brachii muscle, challenging the traditional concept of its exclusive radial nerve supply. Evidence from cadaveric and clinical studies demonstrates frequent contributions from the axillary and ulnar nerves, as well as occasional involvement of the musculocutaneous and ulnar collateral nerves. These anatomical variations carry important clinical and surgical implications, particularly in the assessment of nerve injuries and in the planning of reconstructive and nerve transfer procedures involving the posterior arm. Recognition of such variant innervation patterns is essential to prevent iatrogenic nerve injury and to optimise functional outcomes following surgical intervention. Accordingly, incorporation of these findings into anatomical descriptions and surgical education is necessary to improve both anatomical understanding and clinical practice.
